# Enhanced *Caenorhabditis elegans* Locomotion in a Structured Microfluidic Environment

**DOI:** 10.1371/journal.pone.0002550

**Published:** 2008-06-25

**Authors:** Sungsu Park, Hyejin Hwang, Seong-Won Nam, Fernando Martinez, Robert H. Austin, William S. Ryu

**Affiliations:** 1 Division of Nano Sciences (BK21), Ewha Womans University, Seoul, Korea; 2 Department of Physics, Princeton University, Princeton, New Jersey, United States of America; 3 Lewis-Sigler Institute for Integrative Genomics, Princeton University, Princeton, New Jersey, United States of America; Freie Universitaet Berlin, Germany

## Abstract

**Background:**

Behavioral studies of *Caenorhabditis elegans* traditionally are done on the smooth surface of agar plates, but the natural habitat of *C. elegans* and other nematodes is the soil, a complex and structured environment. In order to investigate how worms move in such environments, we have developed a technique to study *C. elegans* locomotion in microstructures fabricated from agar.

**Methodology/Principal Findings:**

When placed in open, liquid-filled, microfluidic chambers containing a square array of posts, we discovered that worms are capable of a novel mode of locomotion, which combines the fast gait of swimming with the more efficient movements of crawling. When the wavelength of the worms matched the periodicity of the post array, the microstructure directed the swimming and increased the speed of *C. elegans* ten-fold. We found that mutants defective in mechanosensation (*mec-4*, *mec-10*) or mutants with abnormal waveforms (*unc-29*) did not perform this enhanced locomotion and moved much more slowly than wild-type worms in the microstructure.

**Conclusion/Significance:**

These results show that the microstructure can be used as a behavioral screen for mechanosensory and uncoordinated mutants. It is likely that worms use mechanosensation in the movement and navigation through heterogeneous environments.

## Introduction

The nematode *Caenorhabditis elegans*, is a model organism used in a wide range of behavioral studies—from sensory transduction [1] to learning and memory [2]. Determining patterns of movement has been important in the characterization of *C. elegans* behavior, including the studies of chemotaxis, thermotaxis, and mechanosensation [1], [3]. Traditionally these experiments have been done on the smooth surface of agar plates, where the general mechanics of locomotion is well understood [4]. Worms crawl on their sides and move by propagating sinusoidal waves along their body. These waves are produced by a sequence of dorsal-ventral muscular contractions that is opposed by a restoring force provided by the internal hydrostatic pressure of the worm. On agar plates, worms are constrained to the surface of the plate by a thin water layer. The surface tension of this water layer presses the worm to the surface, forming a shallow channel that restricts its body from moving laterally. As the backward wave is propagated along the animal, the lateral restrictions force the body to move tangentially to the radius of curvature of its trajectory. Since each segment of the worm's body is then forced to follow the segment in front of it, the worm produces a sinusoidal pattern as the body follows the oscillatory movement of its head. On laboratory plates, worms typically move without slip, and so speed is equal to the wavelength (∼1 mm) times the oscillation frequency (∼0.5 Hz), which is about 0.5 mm/s.

**Figure 1:**
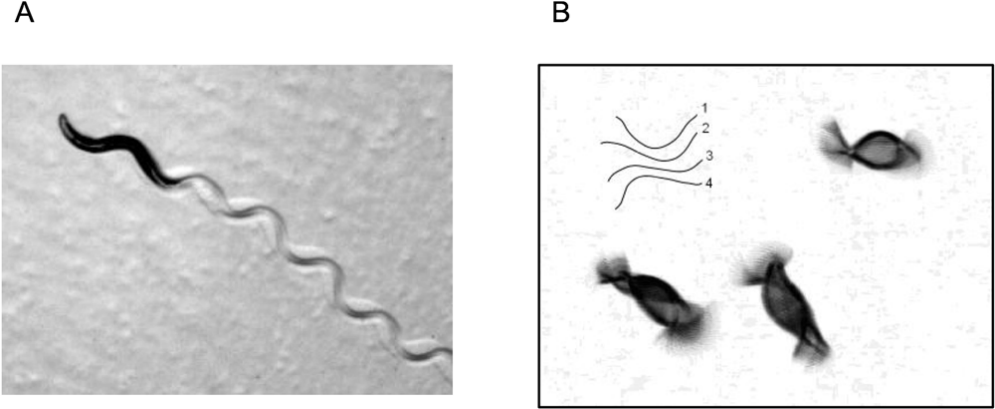
*C. elegans* crawling and swimming. (a) An image of a worm crawling on agar and leaving a sinusoidal trail in a bacterial lawn is shown. Since the worm moves with very little slip, the body follows the curvature of the sinusoidal waves that are propagated down the length of the animal. (b) Multiple exposure of swimming *C. elegans* in bulk liquid. The image shows the variance of a sequence of 20 images taken at 30 Hz of adult N2 worms. Grey scale level of image indicates variance, with high variance (large movements) indicated by black, and zero variance (small movements) indicated by white. Note the bi-nodal bending motions. Inset shows a series of swimming strokes through a half-cycle.

In liquids, swimming motions of worms are fundamentally different than their crawling motions—they move less effectively. Notable differences between swimming versus crawling worms include an increase in oscillation frequency and amplitude, and a decrease in wavelength for swimming worms [5]. Many swimming *C. elegans* produce a bending wave with two distinct nodes (Fig. 1b), a shape similar to the first bending mode of a simple elastic rod. With a body length of about 1 mm and an oscillation frequency of about 1 Hz, the Reynolds number for a worm swimming in water is ∼1, and so viscous and inertial forces are about equal. This makes hydrodynamic analysis of swimming difficult since we cannot make simplifying assumptions of high or low Reynolds number. But the shapes of swimming worms are generally symmetric along the anterior-posterior axis and so it is easy to see qualitatively that these movements would produce little net force (viscous or inertial) to propel the worm forward. Ultimately worms swim with a great deal of slip, moving just a fraction of a wavelength for each oscillatory cycle.

In the wild, worms live in the soil, and so are challenged with a more heterogeneous environment than the smooth agar plates of the laboratory. In order to investigate how worms move through complex and confined spaces—like the interstitial areas between soil particles—we introduced worms into microfluidic structures with features on the spatial scale of the worm. Previous studies have used microfabricated chambers [6], mazes [7], and arrays of posts [8], to explore worm behavior in more structured and challenging environments. However, our structures are unique in that they are cast from agar instead of the elastomer, polydimenthylsiloxane (PDMS), and are left unsealed. This open system is filled with buffer and relies on the surface tension of the liquid surface to keep worms confined. Casting the structures out of agar and leaving them unsealed allows this system to be made more easily and quickly than PDMS structures that require a glass-bonding step, and our open system facilitates loading and retrieving worms from the structure.

To our surprise we found that in a simple structure of uniformly spaced posts, worms are capable of very fast and directed motion. This unusual behavior is a combination of the fast bending motions of swimming worms with the laterally-restricted mechanics of crawling worms. By testing the movements of uncoordinated and mechanosensory defective mutants in the microstructure, we found that this “enhanced swimming” was dependent on the worm's sinusoidal pattern of motion and its mechanosensory feedback.

## Results

To better understand how *C. elegans* swims through heterogeneous environments, we placed worms in microfabricated structures made from 3% agar and recorded their movements with time-lapse video-microscopy. The structures were produced using microlithrographic fabrication techniques previously described [9], but modified to cast agar instead of polydimethylsiloxane. We also cast some of the agar structures from PDMS-based molds, which we found released larger grid structures with fewer defects. The structures consisted of a shallow chamber (20 mm×20 mm×0.11 mm) containing circular posts (300 µm in dia., 0.11 mm tall) in a square grid configuration with a range of center-to-center distances (350 to 550 µm). The chambers were filled to the top with buffer, and worms synchronized for size (900±40 µm in length, 650±40 µm wavelength) were placed in the structures and tested for motility. Worm movements could be studied for several hours.

**Figure 2:**
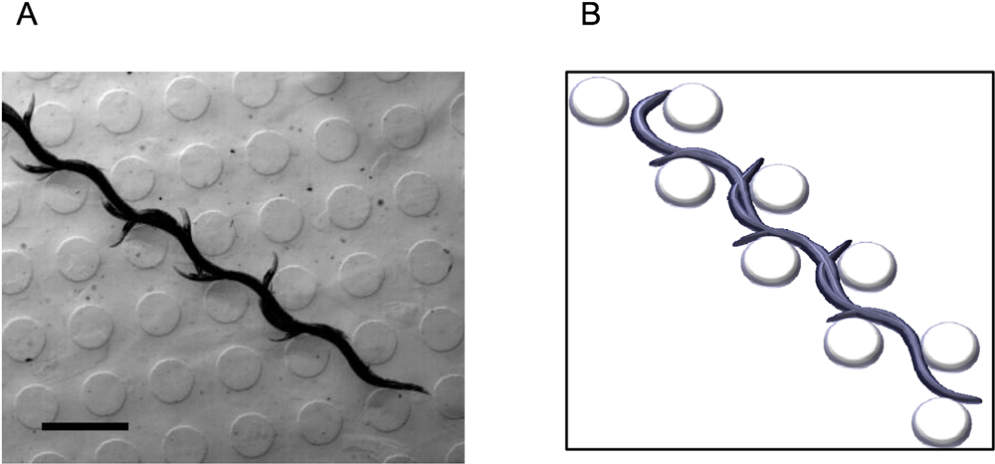
Multiple exposure of a worm performing enhanced swimming in an agar post array. (a) The picture contains 10 time-lapse images taken at 5 Hz, with worms swimming right to left. Post diameter is 300 microns, post spacing is 475 microns center-to-center, and post height is 110 microns. Starting at post 1, worm then contacts post 2 at half cycle, and finally post 3 for a complete cycle. Scale bar = 1 mm. (b) an isometric schematic of the worm motion as it moves in the microfabricated grid.

**Figure 3:**
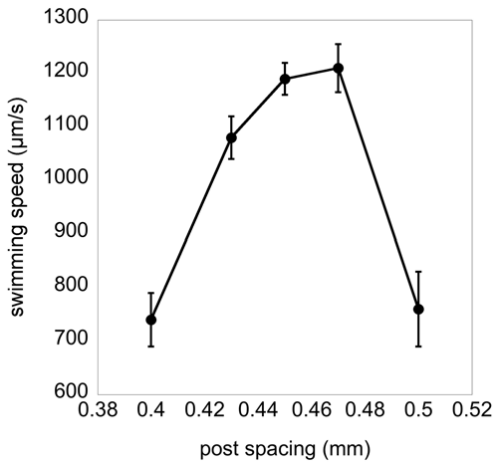
Enhanced swimming speed versus post spacing. The speed of worms moving persistently along the diagonal of the post array as shown in Fig. 3, was calculated by measuring the center of mass of the worm as a function of time and taking the discrete derivative. Error bars indicate the standard error.

**Figure 4:**
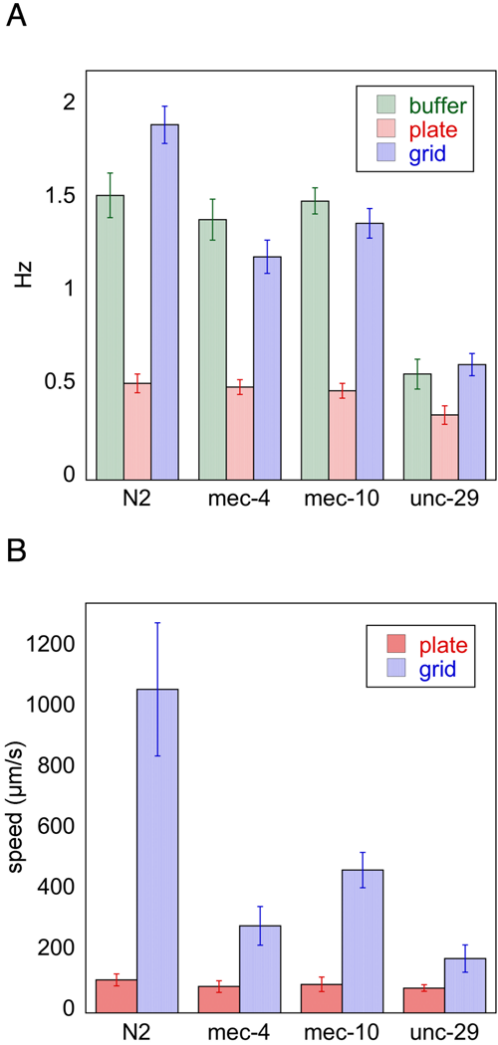
Motility of *C. elegans* strains on agar plates and in the grid microstructures. A) Oscillation frequency of worms in different environments. Error bars indicate the standard deviation. B) Speed of worms only moving in the forward direction (not turning or bending) was measured. For plate measurements *n* = 10, and for grid measurements *n* = 30.

In bulk fluid, worms swam inefficiently with an oscillation frequency of 1.5±0.13 Hz and a velocity of about 0.12±0.03 mm/s. In the microstructures with center-to-center post spacings of 425 to 475 µm, locomotion became strikingly more efficient and directed (Fig. 2, 3). Worms weaved their way through the posts along the diagonal directions *increasing* their swimming bending frequency to 1.92±0.08 Hz (p = 0.001) and reaching speeds greater than 1.3 mm/sec, more than 10× the speed in bulk fluids. This enhanced swimming was persistent and many worms swam from one corner of the structure to the other without interruption. Outside of this range of post spacing (425–475 µm), the probability of enhanced swimming dropped quickly, and the few worms that did perform enhanced swimming swam at a slower speed (Fig. 3). We tested *unc-29* worms which perform slightly abnormal sinusoidal movements [10]—the animals generate a larger amplitude at their heads compared to their tails. These worms move normally on agar with similar oscillation frequency, but when placed in the microfluidic structures they failed to perform enhanced swimming and moved much more slowly in the array (Fig. 4).

**Figure 5:**
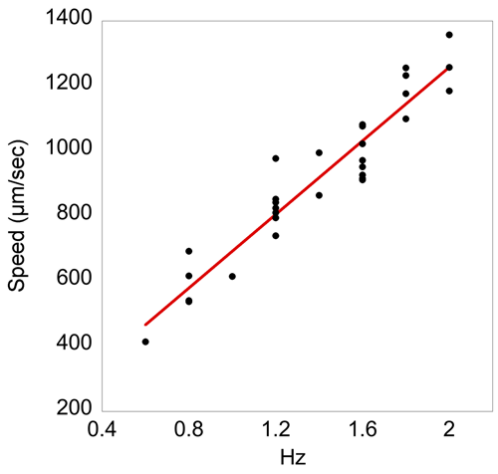
Post-array swimming speed versus oscillation frequency. Speed of N2 worms moving only in the forward direction (not turning or bending) was measured.

We captured and analyzed the motion of worms using computer imaging. It is apparent that the directed motion and increased speed of the worm is the result of the combination of fast oscillations of swimming motility and the reduced slip of crawling mechanics. The liquid environment allows the worm to remain free swimming and the posts allow for an efficient conversion of bending movements to forward motion. Posts spaced approximately at half the wavelength of the worm's sinusoidal waveform provide a point of external force throughout the movement cycle, similar to the lateral restriction provided by the shallow channels that worms make when crawling on the surface of an agar plate. For each half cycle, worms push off posts on either side of its body. After a full cycle, worms have traversed approximately a full wavelength along the diagonal direction (Fig. 2), allowing worms to “swim” with minimal slip. As expected with this mechanism of locomotion, a worm's swimming speed in the post array is linearly proportional to its oscillation frequency (Fig. 5).

*C. elegans* is mechanosensitive [2]. To determine if mechanosensitivity is involved in the enhanced locomotion through the microfluidic structure, we tested a variety of mechanosensory mutants and found that *mec-4*(e1339) and *mec-10*(e1515) showed no differences in movement on agar or in bulk fluids when compared to wild-type worms, but performed at reduce speeds in the grid, failing to sustain the enhanced swimming pattern even though they were the same size as the wild-type worms (Fig. 4). Phenotypically *mec-4* and *mec-10* mutants are defective to “gentle” touches. For example *mec-4* and *mec-10* worms fail to respond to a light touch applied manually with an eyelash, while wild-type worms will respond by accelerating forward or backward depending on the placement of the stimulus. Physiologically MEC-4 and MEC-10 are core subunits of the same mechanosensitive channel [11], [12], and so have a similar mechanosensory defect and produce similar behavior in the microstruture. Apparently, wild-type worms can sense the posts through this “gentle” touch transduction pathway while they are moving through the post array and respond by increasing their oscillation speed. Worms clearly are relying on the transduction of touch in their navigation of the microstructure and probably utilize this sensory pathway more routinely and subtly in their normal movements than we have expected previously.

## Discussion

The understanding of the self-powered motion of organisms through microfabricated structures is the first step towards their use in scientific and in bioengineering applications [13]. Even though the natural environment of *C. elegans* is more complex than our microstructures, we believe that a better understanding of the worm's natural movements can be revealed by studying its behavior in microfabricated structures as described here. We have shown that novel swimming behavior emerges when worms are challenged with an array of posts in a microfluidic environment. This is a surprising mode of locomotion that would not have been discovered under standard laboratory conditions. We also believe that this type of technology will be useful for the basis of behavioral screens.

Previously, microstructures made from PDMS have been used to develop a behavioral assay to screen for oxygen sensing mutants [6] and for olfaction [14]. Other studies have used chambers [6] and maze-like [7] micro-fabricated structures to explore worm behavior, but these devices have larger spatial structures than described here and so worm locomotion was effectively unchanged from movement on agar plates. Our system is similar in design to another post array structure recently used to study worm behavior [8] but presumably their hexagonal-post array does not support the enhanced locomotion we see in our square-post arrays.

Here we show that our simple agar microstructures can differentiate between mechanosensory mutants that show no locomotory defect under normal laboratory conditions. The failure of mechanosensory mutants to navigate the microstructures in the same manner as wild-type worms suggests that mechanosensation is important in how worms navigate the complicated structures of soils. Worms have an exquisite sense of touch, and it has even been shown that worms can “feel” when they are in a lawn of bacteria [15]. It is likely that the worm uses its sense of touch more widely and generally for decisions of navigation and foraging than previously expected. *C. elegans* has likely developed specialized behaviors to deal with heterogeneous environments and so the easy-to-build structured microfluidic environments demonstrated here will be useful in their discovery and study.

## Materials and Methods

### Microfabrication

The grid structures were drawn using L-edit (Tanner Research, Pasadena, CA). The homogeneous grid structures were confined to a square lattice of 20 mm^2^ enclosed by 5 mm thick walls. The pattern of the grid structures was printed on a transparent film using a high-resolution printer with an accuracy of 10 microns. The transparency served as a mask for the subsequent photolithography. The procedure used to fabricate the SU-8 molds has been previously described [9]. Photoresist (SU-8 10, MicroChem, Newton, MA) was spin-coated onto a polished silicon wafer to create a master mold with 110-micron thick features. The spin-coated wafer was exposed to UV light through the mask by a mask aligner (Oriel 8051, Oriel Instruments, Stratford, CT). Unexposed photoresist was removed with SU-8 developer, and the remaining photoresist served as the mold for the agar. 3% Bacto-agar in NGM buffer (50 mM NaCl, 1 mM CaCl_2_, 1 mM MgSO_4_, 25 mM KH_2_PO_4_) was poured at 70°C onto the mold in a petri-dish and left to cool at room temperature. Alternatively, molds made from PDMS were used to cast the agar structure. Air bubbles trapped in the small features of the mold were released by scrapping the surface of the agar with the edge of a razor blade. After 1-hour the agar was cut and lifted from the mold, and placed structure-side up onto a petri-dish.

### Strain preparation and motility assays

The *C. elegans* strain, N2, mec-4, mec-10, and unc-29 were grown at 20°C and maintained under standard conditions [16]. Worms were synchronized using the standard hypochlorite procedure [17]. Just before use, the agar grid structure was filled with NGM buffer. L4 and young adult worms were selected from growth plates with a platinum worm pick, washed in NGM buffer, and placed into the fluid-filled agar microstructure. The top of the structure was left open but worms were confined to the structure by the surface tension of the liquid layer. Deionized water was added to the structure to offset evaporation. For bulk swimming assays, worms were placed in an open petri dish (6 cm dia.) filled with NGM buffer. For crawling assays, single worms were prepared as above but placed on freshly prepared (less than 3 days old) NGM plates.

### Data collection

Movies of worms moving in the microstructures were taken using a stereomicroscope (MZ-APO; Leica Microsystems, Wetzlar, Germany) with an IEEE 1394 CMOS camera (Basler A601; Basler, Exton, PA). Motions of worms were analyzed using ImageJ (National Institutes of Health, Bethesda, MD) or using custom programs written in LabVIEW (National Instruments, Austin, TX).
